# Multicenter study of invasive gastric cancer detected after 10 years of *Helicobacter pylori* eradication in Japan: Clinical, endoscopic, and histopathologic characteristics

**DOI:** 10.1002/deo2.345

**Published:** 2024-03-02

**Authors:** Masaaki Kobayashi, Junko Fujisaki, Ken Namikawa, Shu Hoteya, Akiko Sasaki, Kotaro Shibagaki, Kenshi Yao, Seiichiro Abe, Ichiro Oda, Hiroya Ueyama, Hajime Isomoto, Masanori Ito, Mitsushige Sugimoto, Takashi Kawai, Masaaki Kodama, Kazunari Murakami, Kyoichi Adachi, Nobuyuki Matsuhashi, Ken Ohata, Toshikazu Ushijima, Mototsugu Kato, Shin'ichi Miyamoto, Daisuke Yoshimura, Takashi Yao, Kazuyoshi Yagi, Moriya Iwaizumi, Naomi Uemura

**Affiliations:** ^1^ Division of Gastroenterology Niigata Cancer Center Hospital Niigata Japan; ^2^ Department of Gastroenterology Cancer Institute Hospital Japanese Foundation for Cancer Research Tokyo Japan; ^3^ Department of Gastroenterology Toranomon Hospital Tokyo Japan; ^4^ Gastroenterology Medicine Center Shonan Kamakura General Hospital Kanagawa Japan; ^5^ Department of Endoscopy Shimane University Hospital Shimane Japan; ^6^ Department of Endoscopy Fukuoka University Chikushi Hospital Fukuoka Japan; ^7^ Endoscopy Division National Cancer Center Hospital Tokyo Japan; ^8^ Department of Gastroenterology Juntendo University School of Medicine Tokyo Japan; ^9^ Department of Multidisciplinary Internal Medicine Division of Gastroenterology and Nephrology Tottori University Faculty of Medicine Tottori Japan; ^10^ Department of General Internal Medicine Hiroshima University Hospital Hiroshima Japan; ^11^ Department of Gastroenterological Endoscopy Tokyo Medical University Hospital Tokyo Japan; ^12^ Department of Gastroenterology Faculty of Medicine Oita University Oita Japan; ^13^ Health Center Shimane Environment and Health Public Corporation Shimane Japan; ^14^ Department of Gastrointestinal Endoscopy NTT Medical Center Tokyo Tokyo Japan; ^15^ Institute for Advanced Life Sciences Hoshi University Tokyo Japan; ^16^ National Hospital Organization Hakodate National Hospital Hokkaido Japan; ^17^ Department of Gastroenterology National Hospital Organization Kyoto Medical Center Kyoto Japan; ^18^ Department of Gastroenterology National Hospital Organization Kyushu Medical Center Fukuoka Japan; ^19^ Department of Human Pathology Juntendo University Graduate School of Medicine Tokyo Japan; ^20^ Department of Gastroenterology and Hepatology Uonuma Institute of Community Medicine Niigata University Medical and Dental Hospital Niigata Japan; ^21^ Clinical Laboratories Hamamatsu University School of Medicine Shizuoka Japan; ^22^ Department of Gastroenterology Kohnodai Hospital National Center for Global Health and Medicine Chiba Japan

**Keywords:** endoscopic resection, gastric cancer surveillance, mucosal atrophy, pathological stage, undifferentiated type

## Abstract

**Objectives:**

Gastric cancer can be diagnosed even in patients long after *Helicobacter pylori* eradication. Most cases involve intramucosal lesions; however, some are invasive and require surgery. To clarify appropriate long‐term surveillance methods, this study compared invasive gastric cancer diagnosed ≥10 and <10 years after eradication.

**Methods:**

This retrospective multicenter study included 14 institutions. We included 377 patients with gastric cancer with submucosal or deep invasion after surgical or endoscopic resection. Ordered logistic regression analysis was used to explore the factors contributing to the pathological stage and histological type.

**Results:**

Invasive gastric cancer was detected in 84 patients (Group L) and 293 patients (Group S) ≥10 and <10 years after *H. pylori* eradication, respectively. Endoscopic mucosal atrophy at the time of cancer detection was similar in both groups; 50% of the patients had severe atrophy. Annual endoscopy correlated with early pathological stage (odds ratio [OR] 0.28, 95% confidence interval [CI] 0.14–0.54, *p* < 0.001). Group L exhibited an independent correlation with the advanced pathological stage (OR 2.27, 95% CI 1.06–4.88, *p* = 0.035) and the undifferentiated type (OR 2.12, 95% CI 1.16–3.90, *p* = 0.015). The pure differentiated type and early pathological stage significantly (*p* = 0.001) correlated with severe mucosal atrophy in Group S but not in Group L.

**Conclusions:**

Invasive cancers diagnosed ≥10 years after *H. pylori* eradication were likely to be more malignant in histological type and pathological stage. Gastric cancer surveillance should continue regardless of endoscopic atrophy, particularly ≥10 years after eradication.

## INTRODUCTION

In 2013, the Japanese National Health Insurance approved *Helicobacter pylori* eradication therapy for *H. pylori* gastritis.^1^ Gastric cancer has been diagnosed in patients followed up after *H. pylori* eradication, although *H. pylori* eradication reduces the risk.^2^, ^3^ The incidence of gastric cancers diagnosed ≥10 years after *H. pylori* eradication has increased along with the rise in long‐term post‐eradication cases.^4^, ^5^

Small differentiated early‐stage carcinomas are more common in severe mucosal atrophy,^3^, ^6^ whereas undifferentiated carcinomas are more common in mild or moderate atrophy cases.^5^, ^7^ The risk of diffuse‐type gastric cancer is reportedly greater in the second decade of follow‐up than in the first decade following *H. pylori* eradication.^5^ Even though gastric cancer diagnosis after *H. pylori* eradication takes years, many are differentiated intramucosal carcinomas that can be treated endoscopically.^8^, ^9^ However, some lesions are invasive cancer and require surgery, making them a growing concern.^9^, ^10^, ^11^ No comprehensive studies have examined invasive gastric cancer after *H. pylori* eradication^12^, ^13^; therefore, its characteristics are unknown. An appropriate surveillance method for long‐term post‐*H. pylori* eradication is required.

This multicenter study aimed to clarify the clinical, endoscopic, and histopathologic characteristics of invasive gastric cancer diagnosed ≥10 years after *H. pylori* eradication. We collected cases from participating institutions and compared them with those of invasive gastric cancer diagnosed <10 years after *H. pylori* eradication.

## METHODS

We retrospectively reviewed 377 patients with primary gastric cancer from 14 Japanese institutions who met the inclusion criteria between January 2004 and September 2022. We compared 84 patients with gastric cancers diagnosed ≥10 years after *H. pylori* eradication (Long‐term group; Group L) with 293 patients with gastric cancers diagnosed <10 years after eradication (Short‐term group; Group S).

### Inclusion criteria

1. Surgically or endoscopically resected gastric cancer with submucosal or deeper histological invasion.
2. Patients with histories of *H. pylori* eradication.
3. Confirmed negative results for *H. pylori* infection after eradication.

Successful *H. pylori* eradication therapy was assessed using either the ^13^C‐urea breath test, stool antigen test, culture, or quantification of anti–*H. pylori* Immunoglobulin G levels. Patients with one or more negative results in any of these assays were classified as *H. pylori*–negative. Each institution was confirmed to be negative for *H. pylori* according to the criteria of the respective test method.

### Exclusion criteria

1. Patients with an unknown period of eradication of *H. pylori*.
2. Patients who have undergone gastrectomy in the past.
3. Patients with gastroesophageal junction cancer.
4. Patients with gastric adenocarcinoma of fundic gland type or fundic gland mucosa type.
5. Patients who could not provide informed consent to participate in this study.

Nineteen patients with an unknown period of *H. pylori* eradication (*n* = 10), gastric adenocarcinoma of fundic gland type (*n* = 5), or non‐resection, biopsy‐based diagnosis (*n* = 4) were excluded.

### Study variables

#### Patient data

Age, sex, smoking history, history of gastric or other cancers, family history of first‐degree gastric cancer, disease eligible for *H. pylori* eradication therapy, post–*H. pylori* eradication period and timing of the last endoscopy before gastric cancer detection were retrospectively recorded. We defined annual endoscopy as an endoscopic examination performed within 2 years before the lesion was detected.

#### Histological assessment

According to the Japanese Classification of Gastric Carcinoma, gastric cancer size, macroscopic type, histological type, location, invasive depth, lymphovascular invasion, lymph node metastasis, and distant metastasis were recorded.^14^ Pathological stage was evaluated in surgically resected lesions. Each institution performed pathological diagnosis without a central review. For comparison, the histological type was divided into (1) pure differentiated, (2) differentiated‐predominant mixed, and (3) undifferentiated (pure undifferentiated or undifferentiated‐predominant mixed).

#### Endoscopic assessment of mucosal atrophy

The endoscopic grade of gastric mucosal atrophy was evaluated based on the endoscopic classification system described by Kimura and Takemoto^15^ to classify cases into three grades: mild (C‐1 and C‐2), moderate (C‐3 and O‐1), and severe (O‐2 and O‐3). The endoscopic assessment was performed at gastric cancer diagnosis.

### Ethics

This study was conducted in accordance with the Declaration of Helsinki and approved by the ethical committee of Cancer Institute Hospital, Japanese Foundation for Cancer Research (2021GA1053), and those of each participating institution. The participants provided informed consent using the opt‐out method. Junko Fujisaki was the chief investigator, and Ken Namikawa was in charge of the study secretariat office. The responsible co‐investigator at each participating institution completed the case report forms anonymously and sent them to the study secretariat office, which then compiled and analyzed the study data.

### Statistical analysis

Continuous and categorical variables were expressed as medians (ranges) and numbers (percentages), respectively. Differences in clinicopathological data between the two groups post‐*H. pylori* eradication period and the three groups of mucosal atrophy were evaluated using the Mann–Whitney U test for continuous data and the chi‐squared test for categorical variables. Ordered logistic regression analysis was used to explore the factors contributing to pathological stage and histological type. Odds ratios and 95% confidence intervals were determined. All statistical analyses were performed using SPSS software (v. 27.0; IBM). All reported *p*‐values are two‐sided, with *p* < 0.05 indicating significant differences.

## RESULTS

### Patients’ characteristics

We treated 377 patients with invasive gastric cancer either endoscopically (*n* = 38) or surgically (*n* = 339), including additional surgery after endoscopic resection. We compared 84 patients with gastric cancers diagnosed ≥10 years after *H. pylori* eradication (Group L) with 293 patients with gastric cancers diagnosed <10 years after eradication (Group S; Table 1).

**TABLE 1:** Patients’ characteristics.

	Overall	Group L	Group S	*p‐*value
Age, years, median (range)	70 (38–94)	72 (49–94)	70 (38–91)	0.017
Sex				0.309
Male	288/377 (76.4%)	68/84 (81.0%)	220/293 (75.0%)	
Female	89/377 (23.6%)	16/84 (19.0%)	73/293 (25.0%)	
History of smoking	237/377 (62.9%)	54/84 (64.3%)	183/293 (62.5%)	0.799
Past history of GC	74/368 (20.1%)	13/80 (16.3%)	61/288 (21.2%)	0.430
Past history of other cancer	90/367 (24.5%)	23/81 (28.4%)	67/286 (23.4%)	0.381
Family history of GC	98/317 (30.9%)	22/73 (30.1%)	76/244 (31.1%)	1.000
Diseases eligible for eradication				<0.001
Gastritis	228/377 (60.5%)	38/84 (45.2%)	190/293 (64.8%)	
Gastric ulcer	40/377 (10.6%)	15/84 (17.9%)	25/293 (8.53%)	
Duodenal ulcer	6/377 (1.59%)	1/84 (1.19%)	5/293 (1.70%)	
EMR/ESD of GC	57/377 (15.1%)	9/84 (10.7%)	48/293 (16.4%)	
Others or unknown	46/377 (12.2%)	21/84 (25.0%)	25/293 (8.53%)	
Post‐eradication period, months				Not tested
Median (range)	60 (1–558)	143 (120–528)	49 (1–119)	
<12	22/377 (5.83%)		22/293 (7.51%)	
12 ≤ *X* < 60	141/377 (37.4%)		141/293 (48.1%)	
60 ≤ *X* < 120	130/377 (34.5%)		130/293 (44.3%)	
120 ≤ *X* < 180	67/377 (17.8%)	67/84 (79.8%)		
180 ≤ *X* < 240	13/377 (3.45%)	13/84 (15.5%)		
240≤	4/377 (1.06%)	4/84 (4.76%)		
Last endoscopy before detection				0.060
≤24 months	186/258 (72.1%)	31/51 (60.8%)	155/207 (74.9%)	
24 < *X* ≤ 48 months	40/258 (15.5%)	9/51 (17.6%)	31/207 (15.0%)	
48 months<	32/258 (12.4%)	11/51 (21.6%)	21/207 (10.1%)	
Unknown	119/377 (31.6%)	33/84 (39.3%)	86/293 (29.4%)	0.057

Abbreviations: EMR/ESD, endoscopic mucosal resection/endoscopic submucosal dissection; GC, gastric cancer.

Group L, group of invasive gastric cancers diagnosed ≥10 years after *H. pylori* eradication; Group S, group of invasive gastric cancers diagnosed <10 years after *H. pylori* eradication.

The median ages of these patients were Group L: 72 years (49–94 years) and Group S: 70 years (38–91 years). Patients in Group L were significantly older than those in Group S (*p* = 0.017). No significant differences were observed between Groups L and S in terms of sex, history of smoking, history of gastric or other cancers, or family history of gastric cancer within first‐degree relatives. The diseases eligible for *H. pylori* eradication therapy differed significantly (*p* < 0.001). In Group S, 65% of the patients were treated for *H. pylori* gastritis. The median post‐*H. pylori* eradication was Group L: 143 months (120–528 months), Group S: 49 months (1–119 months). Annual endoscopy was more common in Group S (75%) than in Group L (61%), although the difference was not statistically significant (*p* = 0.060).

### Histopathological findings and endoscopic mucosal atrophy

Table 2 shows the histopathological findings and endoscopic mucosal atrophy. No significant differences were observed between Groups L and S in terms of size, macroscopic type, location, and invasion depth. Regarding the histological type, the undifferentiated type was significantly (*p* = 0.023) more common in Group L (38%) than in Group S (23%). No significant differences were observed between Groups L and S in terms of lymphovascular invasion or lymph node metastasis. However, the incidence of distant metastasis was significantly (*p* = 0.031) higher in Group L (6.7%) than in Group S (1.5%). The pathological stage was more advanced in Group L than in Group S; however, the difference was not statistically significant (*p* = 0.060).

**TABLE 2:** Histopathological findings and endoscopic mucosal atrophy.

	Overall	Group L	Group S	*p‐*value
Size, mm (median, range)	24 (3–170)	23 (3–160)	25 (4–170)	0.310
Macroscopic type				0.760
Type 0‐I, 0‐IIa, 0‐IIb	29/377 (7.69%)	8/84 (9.52%)	21/293 (7.17%)	
Type 0‐IIc, 0‐IIa+IIc	282/377 (74.8%)	61/84 (72.6%)	221/293 (75.4%)	
Type 1, 2, 3, 4, 5	66/377 (17.5%)	15/84 (17.9%)	51/293 (17.4%)	
Location				0.713
Upper	120/377 (31.8%)	28/84 (33.3%)	92/293 (31.4%)	
Middle	140/377 (37.1%)	33/84 (39.3%)	107/293 (36.5%)	
Lower	117/377 (31.0%)	23/84 (27.4%)	94/293 (32.1%)	
Invasive depth				0.256
SM1	137/377 (36.3%)	25/84 (29.8%)	112/293 (38.2%)	
SM2	149/377 (39.5%)	34/84 (40.5%)	115/293 (39.2%)	
MP or deeper	91/377 (24.1%)	25/84 (29.8%)	66/293 (22.5%)	
Histological type				0.023
Pure differentiated	169/377 (44.8%)	29/84 (34.5%)	140/293 (47.8%)	
Differentiated‐predominant mixed	110/377 (29.2%)	24/84 (28.6%)	86/293 (29.4%)	
Undifferentiated	98/377 (26.0%)	31/84 (36.9%)	67/293 (22.9%)	
Lymphovascular invasion	186/366 (50.8%)	42/78 (53.8%)	144/288 (50.0%)	0.700
Lymph node metastasis	68/339 (20.1%)	17/75 (22.7%)	51/264 (19.3%)	0.628
Distant metastasis	9/339 (2.65%)	5/75 (6.67%)	4/264 (1.51%)	0.031
Pathological stage				0.060
IA	227/339 (67.0%)	42/75 (56.0%)	185/264 (70.1%)	
IB	44/339 (13.0%)	14/75 (18.7%)	30/264 (11.4%)	
IIA	17/339 (5.01%)	4/75 (5.33%)	13/264 (4.92%)	
IIB	13/339 (3.83%)	4/75 (5.33%)	9/264 (3.41%)	
IIIA or IIB or IIIC	29/339 (8.55%)	6/75 (8.00%)	23/264 (8.71%)	
IV	9/339 (2.65%)	5/75 (6.67%)	4/264 (1.52%)	
Endoscopic mucosal atrophy^†^				0.608
Mild, C‐1, 2	32/377 (8.49%)	9/84 (10.7%)	23/293 (7.84%)	
Moderate, C‐3, O‐1	157/377 (41.6%)	32/84 (38.1%)	125/293 (42.7%)	
Severe, O‐2, 3	188/377 (49.9%)	43/84 (51.2%)	145/293 (49.5%)	

†Kimura‐Takemoto classification.

Abbreviations: C, closed; O, open.

Group L, Group of invasive gastric cancers diagnosed ≥10 years after *H. pylori* eradication; Group S, Group of invasive gastric cancers diagnosed <10 years after *H. pylori* eradication; SM1, submucosal invasion <0.5mm; SM2, submucosal invasion ≥0.5mm; MP, muscularis propria.

The endoscopic grade of the gastric mucosal atrophy was similar at gastric cancer detection. Approximately half of the patients in both groups had severe gastric atrophy.

### Clinical and histological factors contributing to pathological stage

We analyzed the factors contributing to the pathological stage of all the reviewed patients (Table 3). Size and Group L contributed significantly and independently to a more advanced pathological stage. Annual endoscopy and the pure differentiated type significantly correlated with a less advanced pathological stage.

**TABLE 3:** Ordered logistic regression analysis of pathological stage with clinical and histological factors

	OR	95% CI	*p*‐Value
Size	1.06	1.04–1.07	<0.001
Pure differentiated type	0.31	0.14–0.69	0.004
Annual endoscopy	0.28	0.14–0.54	<0.001
Endoscopic severe mucosal atrophy	0.61	0.31–1.20	0.160
Diagnosis ≥10 years after *H. pylori* eradication	2.27	1.06–4.88	0.035

Abbreviations: CI, confidence interval; OR, odds ratio.

### Clinical and endoscopic factors contributing to histological type

We analyzed the factors contributing to histological type in all the reviewed patients (Table 4). Older age, male sex, and severe endoscopic mucosal atrophy contributed significantly to the pure differentiated type. Group L exhibited a significant and independent correlation with the undifferentiated type.

**TABLE 4:** Ordered logistic regression analysis of histological types with clinical and endoscopic factors.

	OR	95% CI	*p*‐Value
Age	0.96	0.93–0.98	0.003
Male	0.38	0.21–0.69	0.002
Annual endoscopy	0.62	0.36–1.05	0.077
Endoscopic severe mucosal atrophy	0.53	0.32–0.87	0.013
Diagnosis ≥10 years after *H. pylori* eradication	2.12	1.16–3.90	0.015

Abbreviations: CI, confidence interval; OR, odds ratio.

### Association of histopathological findings with endoscopic mucosal atrophy

**FIGURE 1:**
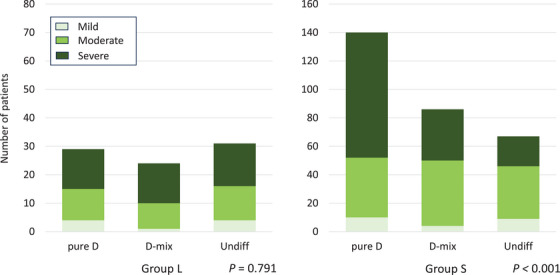
Number of patients with invasive gastric cancer classified as mild, moderate, or severe according to the Kimura and Takemoto classification of endoscopic mucosal atrophy. In Group S, the histological type significantly correlated with the endoscopic grade of mucosal atrophy (*p* < 0.001, Chi‐squared test). However, these correlations were not observed in Group L. Group L included patients with invasive gastric cancers diagnosed ≥10 years after *Helicobacter pylori* (*H. pylori*) eradication, whereas Group S included patients with invasive gastric cancers diagnosed <10 years after *H. pylori* eradication. Pure D, pure differentiated type; D‐mix, differentiated‐predominant mixed type; Undiff, undifferentiated type.

**FIGURE 2:**
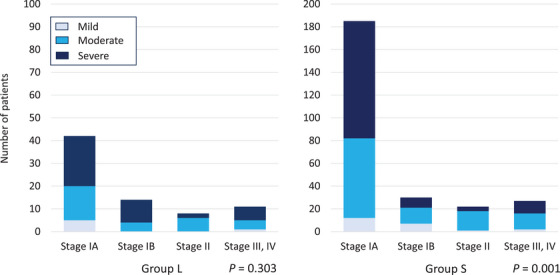
Number of patients with invasive gastric cancer classified as mild, moderate, or severe according to the Kimura and Takemoto classification of endoscopic mucosal atrophy. In Group S, the pathological stage significantly correlated with the endoscopic grade of mucosal atrophy (*p* = 0.001, Chi‐squared test). However, these correlations were not observed in Group L. Group L: Patients with invasive gastric cancers diagnosed ≥10 years after *Helicobacter pylori* (*H. pylori*) eradication; Group S: Patients with invasive gastric cancers diagnosed <10 years after *H. pylori* eradication.

We compared the endoscopic grade of gastric mucosal atrophy with histological types and pathological stages in Groups L and S (Figures 1 and 2). In Group S, the endoscopic grade of mucosal atrophy significantly correlated with the histological type and pathological stage (*p* < 0.001 and *p* = 0.001, respectively). However, these correlations were not observed in Group L. The pure differentiated type was lower in Group L than in Group S, even with severe mucosal atrophy (Figure 1). Similarly, there was no evidence of a higher proportion of stage IA cases with severe or moderate mucosal atrophy in Group L (Figure 2).

## DISCUSSION

This multicenter database analysis revealed the characteristics of patients with invasive gastric cancer after *H. pylori* eradication. Endoscopic or histological findings of early‐stage gastric cancer after *H. pylori* eradication are becoming clearer with more previous reports.^2^, ^3^, ^4^, ^5^, ^6^, ^7^, ^8^, ^9^ After *H. pylori* eradication, few reports of invasive gastric cancer exist,^12^, ^13^ and its characteristics are unknown. To our knowledge, this is the first study to compare the clinicopathological features of invasive gastric cancer diagnosed ≥10 years after *H. pylori* eradication with those detected <10 years after eradication.

Herein, most patients were older men aged ≥70 years and had moderate to severe degrees of endoscopic mucosal atrophy, which is consistent with the findings of previous reports on early‐stage gastric cancer after *H. pylori* eradication. Mucosal atrophy improves with long‐term follow‐up after *H. pylori* eradication.^16^, ^17^ However, at ≥10 years of *H. pylori* eradication, the degree of endoscopic background mucosal atrophy was similar to that observed <10 years of eradication, showing moderate to severe atrophy. These prolonged risks, older men, and endoscopic mucosal atrophy were not reduced by *H. pylori* eradication.

A population‐based prospective cohort study reported the highest risk of gastric cancer in patients with both smoking habits and *H. pylori* infection.^18^ However, few studies have reported an association between smoking and gastric cancer post‐*H. pylori* eradication cases.^19^ In this study, smoking was also strongly associated with gastric cancer in cases that occurred after *H. pylori* eradication cases; however, no significant differences were observed between the status observed ≥10 and that observed <10 years after eradication. Similarly, no differences were found in the history of gastric cancer, other cancers, or family history of gastric cancer. We identified no promising risk factors predicting cancer development 10 years later.

In this study, the pure differentiated type was more common in invasive gastric cancer cases diagnosed <10 years after *H. pylori* eradication than it was in those diagnosed ≥10 years after eradication. The pure differentiated type was also more common in patients with severe mucosal atrophy in invasive gastric cancer diagnosed <10 years after *H. pylori* eradication than it was in those diagnosed ≥10 years after eradication (Figure 1). The pathological stage was also associated with endoscopic mucosal atrophy. More patients with severe mucosal atrophy had pathological stage IA in invasive gastric cancers diagnosed <10 years after *H. pylori* eradication than they did in those diagnosed ≥10 years after eradication (Figure 2).

Invasive gastric cancers diagnosed ≥10 years after *H. pylori* eradication were more often of the undifferentiated or differentiated‐predominant mixed type and had little association with endoscopic mucosal atrophy (Figure 1). The mucosal atrophy did not affect the pathological stage in invasive gastric cancers diagnosed after ≥10 years (Figure 2). Long‐term post‐eradication increases the risk of undifferentiated or differentiated mixed‐type gastric cancer, which is pathologically staged as high‐grade. No studies have reported a higher frequency of undifferentiated intramucosal carcinomas in long‐term post‐eradication patients. Speculations suggest that over the long‐term post‐eradication period, improvement in histological mucosal atrophy may facilitate the development of undifferentiated or differentiated mixed‐type cancers. This study did not histologically examine mucosal atrophy. However, long‐term follow‐up after *H. pylori* eradication may restore histological atrophic changes, even if the endoscopic mucosal atrophy is severe or moderate. Endoscopic atrophy improved less than histological atrophy by 36–153 months (mean, 78 months) after *H. pylori* eradication.^20^

Similar to this study, another study reported that patients with mild to moderate gastric atrophy were at an increased risk of developing diffuse‐type gastric cancer 10–20 years after *H. pylori* eradication.^5^ Although the study design (prospective cohort vs. retrospective case‐control), histological type (diffuse type vs. undifferentiated type), the timing of endoscopic background mucosal assessment (at time of eradication vs. at time of lesion discovery), and main diseases deemed eligible for *H. pylori* eradication (peptic ulcer vs. gastritis or after endoscopic mucosal resection/endoscopic submucosal dissection) were different between the two studies, their results were congruent.

Endoscopic images and histological findings of invasive gastric cancer after *H. pylori* eradication are highly variable, and accurately detecting all lesions is difficult. In the macroscopic type, lesions showing depression were the most common, accounting for 75% of the cases, including IIc‐like advanced cancers that invaded below the proper muscle. The median diameter of the lesion was 24 mm. Regardless of the time of detection, small advanced cancers <30 mm were present (*n* = 17), while relatively large submucosal cancers >50 mm were observed (*n* = 15). In this study, annual endoscopy significantly correlated with the early pathological stage. As 44% (164/377) of invasive cancers were detected within 5 years of *H. pylori* eradication, appropriate examinations must be performed for 5 years or longer following eradication. Endoscopic examination before *H. pylori* eradication cannot be neglected, and the accuracy of the endoscopic examination before and after eradication is also a factor in determining whether gastric cancer can be detected before invasion.

After *H. pylori* eradication, the disease is generally discovered at an invasive stage owing to irregular follow‐up; however, such lesions also develop in patients undergoing annual endoscopy. After *H. pylori* eradication, the endoscopist must detect gastric cancer sufficiently early for endoscopic or curative surgery. Although numerous gastric cancers can occur after *H. pylori* eradication, and each lesion develops differently, we believe that many lesions can be diagnosed at a curable stage with diligent periodic examinations.

This study had some limitations. First, although this study included many patients from 14 institutions, it had a retrospective design. Second, as time passes after *H. pylori* eradication, it becomes increasingly difficult for patients to recall the reason for eradication and to continue periodic examinations. Third, as the participating institutions were specialized oncology hospitals, the frequency of referred patients was high. The assessment of *H. pylori* infection is very important after eradication; however, it was sometimes performed by referring physicians using methods other than the recommended ^13^C‐urea breath test or stool antigen test. Finally, histological types may change before and after cancer invasion. Therefore, we have planned a separate study to determine the frequency of undifferentiated types, including intramucosal cancers.

In conclusion, invasive gastric cancers diagnosed ≥10 years after *H. pylori* eradication are often undifferentiated types. Irrespective of endoscopic mucosal atrophy, these tumors were more biologically malignant in terms of their histological types and pathological stages. Thus, whether this was caused by a delayed diagnosis or histological restoration of mucosal atrophy requires further investigation. In clinical practice, gastric cancer surveillance should be continued regardless of endoscopic atrophy, particularly after 10 years of *H. pylori* eradication.

## CONFLICT OF INTEREST STATEMENT

Seiichiro Abe is an associate editor of *DEN Open*. The other authors declare no conflict of interest.

## References

[1] Tsuda M , Asaka M , Kato M . Effect on *Helicobacter pylori* eradication therapy against gastric cancer in Japan. Helicobacter 2017; 22: e12415. 28771894 10.1111/hel.12415 PMC5655764

[2] Kamada T , Hata J , Sugiu K *et al.* Clinical features of gastric cancer discovered after successful eradication of *Helicobacter pylori*: Results from a 9‐year prospective follow‐up study in Japan. Aliment Pharmacol Ther 2005; 21: 1121–1126. 15854174 10.1111/j.1365-2036.2005.02459.x

[3] Fukase K , Kato M , Kikuchi S *et al.* Effect of eradication of *Helicobacter pylori* on incidence of metachronous gastric carcinoma after endoscopic resection of early gastric cancer: An open‐label, randomised controlled trial. Lancet 2008; 372: 392–397. 18675689 10.1016/S0140-6736(08)61159-9

[4] Take S , Mizuno M , Ishiki K *et al.* Seventeen‐year effects of eradicating *Helicobacter pylori* on the prevention of gastric cancer in patients with peptic ulcer; A prospective cohort study. J Gastroenterol 2015; 50: 638–644. 25351555 10.1007/s00535-014-1004-5

[5] Take S , Mizuno M , Ishiki K *et al.* Risk of gastric cancer in the second decade of follow‐up after *Helicobacter pylori* eradication. J Gastroenterol 2020; 55: 281–288. 31667586 10.1007/s00535-019-01639-w PMC7026240

[6] Yamamoto K , Kato M , Takahashi M *et al.* Clinicopathological analysis of early‐stage gastric cancers detected after successful eradication of *Helicobacter pylori* . Helicobacter 2011; 16: 210–216. 21585606 10.1111/j.1523-5378.2011.00833.x

[7] Tanaka M , Hoteya S , Kikuchi D *et al.* Effect of *Helicobacter pylori* infection on malignancy of undifferentiated‐type gastric cancer. BMC Gastroenterol 2022; 22: 7. 34991485 10.1186/s12876-021-02034-7 PMC8734290

[8] Mori G , Nakajima T , Asada K *et al.* Incidence of and risk factors for metachronous gastric cancer after endoscopic resection and successful *Helicobacter pylori* eradication: Results of a large‐scale, multicenter cohort study in Japan. Gastric Cancer 2016; 19: 911–918. 26420267 10.1007/s10120-015-0544-6

[9] Hata K , Ito M , Boda T *et al.* Gastric cancer with submucosal invasion after successful *Helicobacter pylori* eradication: A propensity score‐matched analysis of patients with annual patient endoscopic survey. Digestion 2019; 99: 59–65. 30554223 10.1159/000494414

[10] Kodama M , Mizukami K , Hirashita Y *et al.* Differences in clinical features and morphology between differentiated and undifferentiated gastric cancer after *Helicobacter pylori* eradication. PLoS One 2023; 18: e0282341. 37000845 10.1371/journal.pone.0282341 PMC10065271

[11] Suzuki H , Nonaka S , Maetani I *et al.* Clinical and endoscopic features of metachronous gastric cancer with possible lymph node metastasis after endoscopic submucosal dissection and *Helicobacter pylori* eradication. Gastric Cancer 2023; 26: 743–754. 37160633 10.1007/s10120-023-01394-1

[12] Tanaka M , Kikuchi D , Odagiri H *et al.* Advanced gastric cancer detected during regular follow‐up after eradication of *Helicobacter pylori* . Clin J Gastroenterol 2022; 15: 358–362. 35020137 10.1007/s12328-021-01577-2

[13] Tokura J , Namikawa K , Nakano K *et al.* Clinicopathological characteristics of advanced gastric cancer after *Helicobacter pylori* eradication. JGH Open 2022; 6: 833–838. 36514501 10.1002/jgh3.12827 PMC9730718

[14] Japanese Gastric Cancer Association . Japanese classification of gastric carcinoma: 3rd English edition. Gastric Cancer 2011; 14: 101–112. 21573743 10.1007/s10120-011-0041-5

[15] Kimura K , Takemoto T . An endoscopic recognition of the atrophic border and its significance in chronic gastritis. Endoscopy 1969; 3: 87–97.

[16] Kodama M , Murakami K , Okimoto T *et al.* Ten‐year prospective follow‐up of histological changes at 5 points on the gastric mucosa as recommended by the updated Sydney system after *Helicobacter pylori* eradication. J Gastroenterol 2012; 47: 394–403. 22138891 10.1007/s00535-011-0504-9

[17] Kodama M , Okimoto T , Mizukami K *et al.* Gastric mucosal changes, and sex differences therein, after *Helicobacter pylori* eradication: A long‐term prospective follow‐up study. J Gastroenterol Hepatol 2021; 36: 2210–2216. 33656793 10.1111/jgh.15477

[18] Shikata K , Doi Y , Yonemoto K *et al.* Population‐based prospective study of the combined influence of cigarette smoking and *Helicobacter pylori* infection on gastric cancer incidence: The Hisayama study. Am J Epidemiol 2008; 168: 1409–1415. 18945691 10.1093/aje/kwn276

[19] Hatta W , Koike T , Asonuma S *et al.* Smoking history and severe atrophic gastritis assessed by pepsinogen are risk factors for the prevalence of synchronous gastric cancers in patients with gastric endoscopic submucosal dissection: A multicenter prospective cohort study. J Gastroenterol 2023; 58: 433–443. 36786863 10.1007/s00535-023-01967-y

[20] Kodama M , Okimoto T , Ogawa R *et al.* Endoscopic atrophic classification before and after *H. pylori* eradication is closely associated with histological atrophy and intestinal metaplasia. Endosc Int Open 2015; 03: E311–E317. 10.1055/s-0034-1392090 PMC4554494 26357676

